# Soluble Fibrinogen Triggers Non-cell Autonomous ER Stress-Mediated Microglial-Induced Neurotoxicity

**DOI:** 10.3389/fncel.2018.00404

**Published:** 2018-11-19

**Authors:** Thomas M. Piers, Emma East, Claudio Villegas-Llerena, Ioanna G. Sevastou, Mar Matarin, John Hardy, Jennifer M. Pocock

**Affiliations:** ^1^Cell Signalling Laboratory, Department of Neuroinflammation, Institute of Neurology, University College London, London, United Kingdom; ^2^Department of Molecular Neuroscience, Institute of Neurology, University College London, London, United Kingdom; ^3^Department of Neuropsychology, National Hospital for Neurology and Neurosurgery, University College London Hospitals, London, United Kingdom

**Keywords:** microglia, neurodegeneration, Alzheimer’s disease, ER stress, fibrinogen

## Abstract

Aberrant or chronic microglial activation is strongly implicated in neurodegeneration, where prolonged induction of classical inflammatory pathways may lead to a compromised blood-brain barrier (BBB) or vasculature, features of many neurodegenerative disorders and implicated in the observed cognitive decline. BBB disruption or vascular disease may expose the brain parenchyma to “foreign” plasma proteins which subsequently impact on neuronal network integrity through neurotoxicity, synaptic loss and the potentiation of microglial inflammation. Here we show that the blood coagulation factor fibrinogen (FG), implicated in the pathogenesis of dementias such as Alzheimer’s disease (AD), induces an inflammatory microglial phenotype as identified through genetic microarray analysis of a microglial cell line, and proteome cytokine profiling of primary microglia. We also identify a FG-mediated induction of non-cell autonomous ER stress-associated neurotoxicity via a signaling pathway that can be blocked by pharmacological inhibition of microglial TNFα transcription or neuronal caspase-12 activity, supporting a disease relevant role for plasma components in neuronal dysfunction.

## Introduction

Midlife elevated plasma levels of systemic inflammatory markers such as fibrinogen and albumin are correlated with reduced brain volumes in brain areas associated with dementia such as Alzheimer’s disease (AD) in later life (Walker et al., 2017). Blood brain barrier (BBB) and cerebral vascular integrity can weaken in normal aging and in neurodegenerative diseases, and may contribute to cognitive decline (Hainsworth et al., 2017; Sweeney et al., 2018). Deposition of blood-borne proteins in the CNS parenchyma may occur in neurodegenerative diseases such as AD (Lipinski and Sajdel-Sulkowska, 2006; Ryu and McLarnon, 2009; Miners et al., 2018) and depletion of blood-borne factors, specifically fibrinogen (FG) are protective in animal models of neurodegeneration (Cortes-Canteli et al., 2012).

Fibrinogen may enter the brain via small cerebral microbleeds or microhaemorrhages (Werring et al., 2010), which are associated with the development of depressive symptoms in AD (Leeuwis et al., 2017), an often early event in AD (Cortés et al., 2018), and the inflammatory activation of microglia (Ahn et al., 2018). In line with this, infiltration of plasma proteins into the CNS precipitates neurotoxic signaling cascades through the activation of microglia (Huang et al., 2008; Hooper et al., 2009; Ryu and McLarnon, 2009; Weinstein et al., 2009). Furthermore, systemic inflammation is correlated with future cognitive decline in AD (Holmes et al., 2009). Moreover FG can induce pro-inflammatory cytokine secretion from peripheral blood cells (Jensen et al., 2007). In addition, elevated plasma FG levels are a risk factor for AD and vascular dementia, (van Oijen et al., 2005; Xu et al., 2008), a proposed CFS biomarker for AD (Lee et al., 2007) and the development of cognitive impairment after stroke (Gillett et al., 2018).

Fibrinogen signals through an integrin class of receptor, Mac-1 (or αMβ2, CD11b/CD18), expressed by microglia and macrophages (Adams et al., 2007) which is upregulated in AD brain (Akiyama and McGeer, 1990). This signaling stimulates the secretion of pro-inflammatory cytokines and chemokines (Smiley et al., 2001; Piers et al., 2011). Thus, it seems plausible that FG-induced deficits in neuronal viability are due to aberrant inflammatory signaling. Historically, investigations into chronic, aberrant neuronal loss have focussed on classic apoptotic pathways centered on mitochondrial signaling. However, an endoplasmic reticulum (ER) centered hypothesis for the neuronal death occurring in neurodegenerative diseases has also been proposed (Lindholm et al., 2006). ER stress is a well-characterized cellular phenomenon, whereby an accumulation of misfolded proteins and calcium dys-homeostasis lead to disturbances in the structure and function of the ER (Gerakis and Hetz, 2018). Under normal circumstances, highly conserved unfolded protein response (UPR) adaptive mechanisms rebalance cellular homeostasis. However, if the accumulation of misfolded proteins is prolonged, as observed in AD models (Nakagawa et al., 2000), apoptotic cell signaling cascades are induced, leading to significant cytotoxicity (Nakagawa et al., 2000; Gerakis and Hetz, 2018; Morris et al., 2018). Interestingly, increased pro-inflammatory cytokine release, specifically tumor necrosis factor-α (TNFα) and interleukin-6 (IL-6), can induce ER stress signaling in fibroblast cell lines and *in vivo* (Xue et al., 2005; Denis et al., 2010; O’Neill et al., 2013). Conversely, ER stress can also mediate upregulation of pro-inflammatory cytokines (Chen et al., 2013), suggesting the possibility of a self-perpetuating mechanism of detrimental signaling in neurodegenerative diseases centered on ER stress and aberrant, or chronic, inflammatory signaling. With this in mind, we aimed to identify if exposure to FG modulated inflammatory signaling and subsequent neuronal toxicity in a well-characterized *in vitro* neuronal cell culture model, which can be pharmacologically manipulated to eliminate the presence of microglia (Piers et al., 2011; Jebelli et al., 2015). Here, we identify for the first time, a FG-induced increase in neurotoxicity via a non-cell autonomous mechanism involving a microglial TNFα-mediated induction of neuronal ER stress signaling.

## Materials and Methods

### Materials

Fibrinogen, lipopolysaccharide from *Escherichia coli* (055:B5; LPS), tunicamycin, thapsigargin, leucine-methyl-ester (LME), staurosporine, hirudin, and Isolectin B_4_ from *Griffonia simplicifolia*-FITC conjugated were from Sigma (Dorset, United Kingdom) and thalidomide was from Tocris Bioscience (Bristol, United Kingdom). Quantikine M TNFα. TGF-β and IL-6 ELISA kits, z-VAD-FMK and z-ATAD-FMK were from R&D Systems (Abingdon, United Kingdom). Caspase 12 (FITC-ATAD-FMK) activity kits were from Promokine (Heidelberg, Germany) and caspase 3/7 (FAM-DEVD-FMK) activity kits were from Millipore (Watford, United Kingdom). Anti-ED1, anti-CD11b and rat IgG isotype controls were from AbD Serotec (Kidlington, United Kingdom), anti-caspase 12 and goat anti-rat-FITC were from Abcam (Cambridge, United Kingdom). Goat anti-rabbit IgG horseradish peroxidase (HRP) and goat anti-mouse IgG HRP were from Autogen Bioclear (Calne, United Kingdom).

### Methods

#### BV2 Microglial Cell Line Culture

BV2 microglia were maintained in DMEM with 10% foetal bovine serum (FBS), supplemented with 2 mM L-glutamine, 100 U/ml penicillin and 100 mg/ml streptomycin at 37°C in a humidified atmosphere with 5% CO_2_. Prior to experimentation, BV2 microglia were harvested and plated in serum-free DMEM.

#### Preparation of Enriched Neuronal-Glial Cultures (ENGC)

Primary cultures of enriched neuronal-glial cerebellar cultures were isolated from 3 to 6 day-old Sprague Dawley rat pups and prepared as described previously (Piers et al., 2011) in accordance with the United Kingdom Animals (Scientific Procedures) Act, 1986. Cells were plated on 13 mm poly-D-lysine (PDL) coated glass coverslips at a density of 8 × 10^5^ per coverslip and maintained in ENGC medium (Neurobasal medium with 2% B27 neuronal supplement, 20 mM KCl, 6 g/l D-Glucose, 2 mM L-Glutamine, 50 U/ml penicillin, and 50 μg/ml streptomycin). After 36 h *in vitro*, cytosine furanoarabinoside (10 μM) was added to prevent proliferation of non-neuronal cells. The cultures were maintained at 37°C in 5% CO_2_ and used at 7 DIV.

#### Microglial Ablation From ENGCs

Microglia present in the ENGCs were depleted with 25 mM leucine-methyl-ester (LME), as previously described (Jebelli et al., 2015).

#### Preparation of Primary Cultured Microglia

Microglia were isolated from 5-day-old Sprague Dawley rat pups, as previously described (Piers et al., 2011) in accordance with the United Kingdom Animals (Scientific Procedures) Act, 1986. Cells were plated at a density of 5 × 10^4^/well on 13 mm coverslips or 1 × 10^6^/well in 60 mm culture dishes. After 24 h *in vitro*, the medium was replaced with serum-free ENGC medium and the cultures were left to rest for at least 3 h prior to stimulation.

#### Fibrinogen Preparation

Fibrinogen was prepared in ultrapure, endotoxin-free water at 1000 × final concentration and sonicated prior to addition to cell cultures. The final concentrations used are in line with those previously used *in vitro* (Schachtrup et al., 2010).

#### Microarray Analyses

Extracted RNA from control and FG-treated BV2 cultures (miRNeasy; Qiagen) was sent to AROS Applied Biotechnology (Denmark) for microarray analysis using a MouseRef-8 v2.0 Expression Array (Illumina). Experimental procedures (cDNA labeling and hybridization) were performed according to manufacturer’s instructions. Bead Arrays were scanned using the Illumina Bead Station 500X, and raw intensity values were saved in Illumina’s Bead Studio program manager. For array hybridization, all samples were distributed among different arrays to minimize batch effect. Analysis was performed using Partek Genomics Suite 6.6 (Partek, Inc., St. Louis, MO, United States) and Lumi R package (Bioconductor, Du et al., 2008). Raw expression data were log2 transformed, and all samples were quantile normalized. QC plots before and after normalization were generated for data quality analysis. Individual probes were excluded from analysis if the detection *p*-value was >0.05 in more than 2 out of the 3 repeats for any condition. Samples were also excluded if <95% of the probes were detected (all samples met this quality control criterion). A conservative statistical threshold of FDR <0.05 and minimum fold difference ≥1.5 between sample groups was used in all comparisons and to generate gene lists.

#### Assessment of Cell Death

Live ENGCs were incubated with propidium iodide (PI; 5 μg/ml) and Hoechst 33342 (5 μg/ml). Using Image J analysis software (NIH, United States), dead cells (PI ^+^) were quantified as a percentage of total cell number, the latter obtained by Hoechst 33342 staining. A minimum of 3 fields per coverslip were counted, and at least 3 coverslips were assessed for each variable per experiment in at least 3 separate experiments as previously described (Piers et al., 2011).

#### Immunocytochemistry

Cultures were fixed in 4% paraformaldehyde (PFA) in phosphate buffered saline (PBS) then permeabilised in 100% methanol. Cells were blocked with PBS containing 4% normal goat serum and then incubated with primary antibodies overnight at 4°C (1:100 anti-ED1, 1:100 anti-CD11b, or anti-rat IgG isotype control). After washing, cells were incubated with goat anti-rat FITC (1:100) in PBS. For microglia identification, FITC conjugated lectin (IB_4_) (Streit, 1990) was administered at the same time as the secondary antibodies. Cultures were washed and incubated with DAPI solution prior to mounting with Vectashield (Vector Labs Inc, Burlingame, CA, United States).

#### Assessment of Caspase 12 or Caspase 3/7 Induction

ENGCs were assessed for the induction of caspase-12 and caspase-3/7 after treatment as per the manufacturer’s instructions. After treatment, coverslips were removed and mounted into a basic medium consisting of 153 mM NaCl, 3.5 mM KCl, 0.4 mM KH_2_PO_4_, 20 mM N-Tris (hydroxymethyl)methyl-2-aminoethanesulphonic acid (TES), 5 mM NaHCO_3_, 1.2 mM Na_2_SO_4_, 1.2 mM MgCl_2_, 1.3 mM CaCl_2_, 5 mM glucose, warmed to 37°C and observed immediately using fluorescence microscopy (Zeiss, Oberkochen, Germany). Cultures were counterstained with Hoechst for total cell number and PI for total cell death using automatic exposure settings. A minimum of 3 fields per coverslip was counted, and at least 4 coverslips were assessed for each treatment per experiment. Manual exposure settings were optimized against a positive control then fixed for entire experiments. Analysis was performed using Image J software (NIH, United States).

#### Western Blotting

Soluble protein lysates were resolved by SDS-PAGE electrophoresis and transferred to PVDF membranes using standard techniques. The following antibodies were used: anti-caspase 12 (Abcam; 1:1000), anti-β-actin (Sigma; 1:10,000). Immunoreactive bands were imaged and optical densities were quantified using ImageJ software (NIH, United States) and normalized to β-actin protein levels.

#### ELISA Determination of TNFα, TGF-β1 and IL-6

Quantification of secreted inflammatory cytokines TNFα, IL-6 and TGF-β1 in microglial and ENGC culture supernatants was determined with Quantikine M Rat Immunoassay kits according to the manufacturer’s instructions. Briefly, microglial-conditioned medium or ENGC-conditioned medium (MGCM or ENGC-CM, respectively) was centrifuged at 4500 × *g* for 1 min to remove any floating cells and assayed essentially as previously described (Taylor et al., 2005). Cytokine concentrations were determined against a standard concentration curve.

#### Cytokine Arrays and Pro-inflammatory Cytokine Panel

MGCM was collected as above for cytokine analysis, supernatants pooled from 3 independent experiments and incubated with Proteome ProfilerTM Rat Cytokine array membranes (R&D systems Panel A) as per the manufacturer’s instructions. Data were analyzed using the Protein Array Analyser Palette plugin (ImageJ), and plotted, as a mean ± SEM after normalizing to membrane reference positive controls and intracellular protein concentrations.

### Statistical Analysis

All Western blots and PCR analyses were carried out at least three times and those shown are representative. All experiments were performed from at least three separate cell preparations with internal replicates of at least 3 per experiment. Significant differences were estimated using Welch’s two-sided *t*-test or Student’s un-paired *t*-test with levels of significance at ^∗∗∗^*p* < 0.001, ^∗∗^*p* < 0.01, and ^∗^*p* < 0.05; *p* > 0.05 was not significant.

## Results

### FG Exposure Enhances Inflammatory Gene Expression and Release of Pro-inflammatory Cytokines

**FIGURE 1:**
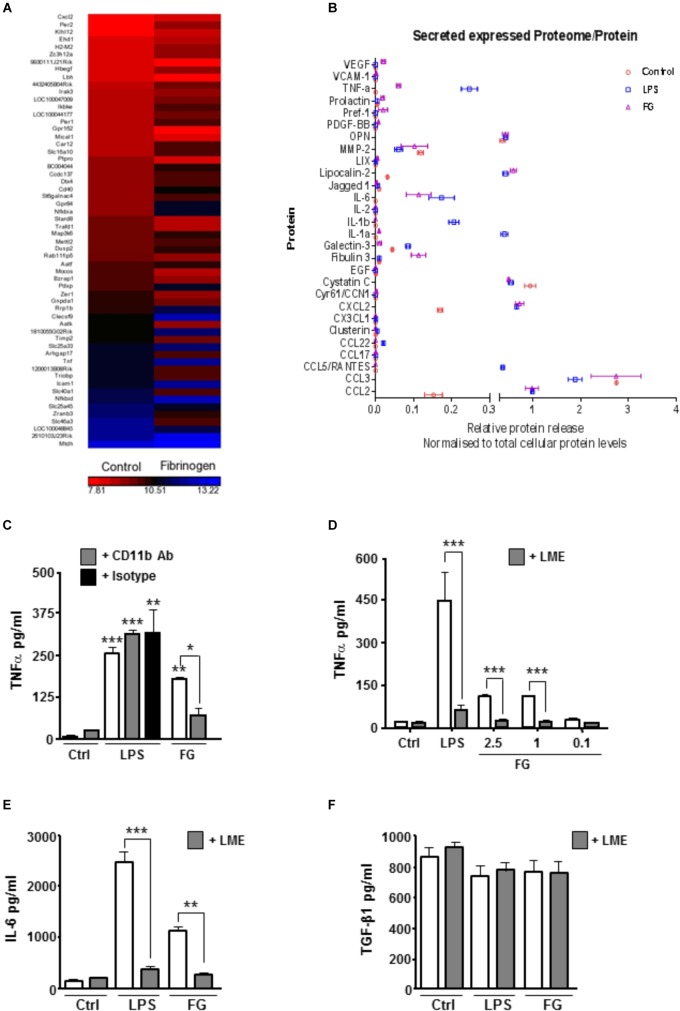
FG induces early upregulation of inflammatory related genes and release of inflammatory cytokines. **(A)** Gene microarray performed on the BV2 microglial cell line after Fibrinogen (2.5 mg/ml) exposure for 6 h compared with non-stimulated (Control) BV2 cells at 6 h. **(B)** Proteome array performed on primary microglia following 24 h of stimulation with lipopolysaccharide (LPS, 1 μg/ml), Fibrinogen (FG, 2.5 mg/ml) or non-stimulated cells (Control). **(C)** TNFα secretion in cell culture medium by ELISA following treatment of primary microglia for 24 h with LPS (1 μg/ml), FG (2.5 mg/ml) or non-stimulated (Ctrl) ± MAC-1 receptor blocking antibody ( + CD11b, 10 μg/ml) or rat IgG as isotype control ( + IgG, 10 μg/ml). **(D)** ENGCs treated for 24 h with LPS (1 μg/ml), FG (0.1–2.5 mg/ml) or non-stimulated (Ctrl), and where indicated, pre-treated with 25 mM leucine-methyl-ester ( + LME) for microglia ablation, followed by ELISA analysis of TNFα secretion in culture medium. **(E)** ENGCs treated as **(D)** followed by ELISA analysis of IL-6 secretion in culture medium. **(F)** ENGCs treated as **(D)** followed by ELISA analysis of TGFβ secretion in culture medium. In all graphs, data are the mean ± SEM from 3 independent experiments with internal replicates of at least 3. Significance levels compared with control condition unless otherwise indicated, ^∗^*p* < 0.05, ^∗∗^*p* < 0.01, ^∗∗∗^*p* < 0.001.

To extend previous findings that FG can induce an inflammatory microglial phenotype, we performed, in parallel, genetic microarray analysis of the well-characterized BV2 mouse microglia cell line, and pro-inflammatory cytokine release from primary rat microglia cultures and enriched neuronal glial cultures (ENGCs). Microarray analysis of BV2 cultures after 6 h of FG exposure identified a significant over-representation, amongst others, of immune system and inflammatory gene upregulation, including *tnf*, which encodes TNFα (Figure 1A and Table 1). These findings were supported by cytokine release data from rat microglia cultures where primary microglia exposed to FG or lipopolysaccharide (LPS; as a positive control) for 24 h showed significant TNFα secretion (Figure 1B). A number of cytokines and chemokines, many of which are regarded as inflammatory (Owen et al., 2011), were secreted to significance by FG (Figure 1B) including TNFα, MMP-2, IL-6, CXCL2, and CCL2. Whilst many mirrored the release evoked with LPS, Fibulin-3 was secreted by FG stimulation only, whereas IL-1β or IL-1α secretion was observed after LPS treatment, but was absent in FG-treated cultures indicating that FG stimulation of microglia is nuanced compared with LPS stimulation.

**Table 1:** PANTHER over-representation analysis of fibrinogen regulated genes.

PANTHER GO-Slim Biological Process	Mus musculus – REFLIST (22320)	FG regulated genes (98)	FG regulated genes (expected)	FG regulated genes (over/under)	FG regulated genes (fold enrichment)	(*P*-value)
Phosphate-containing compound metabolic process (GO:0006796)	931	36	4.09	+	>5	2.92E-22
Catabolic process (GO:0009056)	405	27	1.78	+	>5	6.23E-22
Nitrogen compound metabolic process (GO:0006807)	1064	29	4.67	+	>5	2.50E-13
Localization (GO:0051179)	2788	42	12.24	+	3.43	1.66E-11
Transport (GO:0006810)	2658	41	11.67	+	3.51	1.76E-11
Coenzyme metabolic process (GO:0006732)	97	9	0.43	+	>5	1.36E-07
Metabolic process (GO:0008152)	8467	67	37.18	+	1.8	2.26E-07
**Immune system process (GO:0002376)**	**1480**	**25**	**6.5**	**+**	**3.85**	**8.65E-07**
Cellular process (GO:0009987)	7033	59	30.88	+	1.91	1.04E-06
acyl-CoA metabolic process (GO:0006637)	31	6	0.14	+	>5	1.50E-06
Fatty acid beta-oxidation (GO:0006635)	32	6	0.14	+	>5	1.80E-06
Primary metabolic process (GO:0044238)	6997	57	30.72	+	1.86	8.69E-06
Response to toxic substance (GO:0009636)	55	6	0.24	+	>5	4.29E-05
Nucleobase-containing compound metabolic process (GO:0006139)	3425	35	15.04	+	2.33	1.32E-04
Antigen processing and presentation (GO:0019882)	77	6	0.34	+	>5	2.99E-04
Generation of precursor metabolites and energy (GO:0006091)	290	9	1.27	+	>5	1.30E-03
Fatty acid metabolic process (GO:0006631)	252	8	1.11	+	>5	3.72E-03
Extracellular transport (GO:0006858)	121	6	0.53	+	>5	3.85E-03
Lipid metabolic process (GO:0006629)	966	15	4.24	+	3.54	4.67E-03
Tricarboxylic acid cycle (GO:0006099)	21	3	0.09	+	>5	2.62E-02


In support of previously published data identifying microglial CD11b as a putative receptor for FG (Adams et al., 2007), pre-incubation of primary microglial cultures with a CD11b blocking antibody significantly attenuated the FG-induced TNFα release, but had no effect on LPS-induced secretion (Figure 1C). Given the perceived importance of microglia in mediating the inflammatory effects of FG, and the potential for this inflammation to impact on neuronal integrity, we determined if the microglia present in ENGCs responded in a similar way to isolated primary microglia. The ability to specifically ablate microglia from the ENGCs (Jebelli et al., 2015) allows us to determine any particular dependencies on these cells. In accord with the data for proteome analysis for release of cytokines from primary cultured microglia, exposure of ENGCs to either FG or LPS significantly enhanced the release of TNFα (Figure 1D) and another pro-inflammatory cytokine, interleukin-6 (IL-6; Figure 1E), in a manner similar to that observed in primary microglia cultures. Critically, when microglia were ablated from ENGCs, the LPS- or FG-induced TNFα release (Figure 1C) and IL-6 release (Figure 1D) were significantly attenuated. Further characterization of cytokine release in the ENGCs revealed that transforming growth factor-β1 (TGF-β1) secretion, a cytokine known to have opposing cellular effects to TNFα (Bitzer et al., 2000), was not significantly modulated by either LPS or FG exposure, irrespective of the presence of microglia (Figure 1E), this is possibly due to the dominating expression of this cytokine by the astrocyte population present in the ENGCs (De Groot et al., 1999). Taken together, these data suggest that FG exposure skews microglia toward a pro-inflammatory phenotype.

### FG Induced a Reactive Microglial Phenotype

**FIGURE 2:**
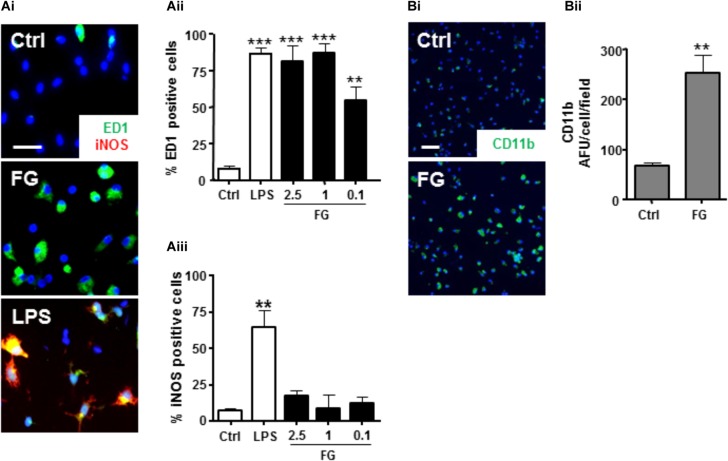
FG induces microglial reactivity. (A) Control primary rat microglia (Ctrl) or microglia exposed to FG (2.5 mg/ml) or LPS (1 μg/ml) for 24 h followed by immunocytochemistry for ED1 and iNOS (Scale bar: 30 μm; **Ai**), and nuclear co-staining with DAPI (blue), quantified as a % percentage of cells expressing ED1 **(Aii)**, or iNOS **(Aiii)**. **(B)** Immunofluorescence of surface CD11b staining in primary microglial cultures following 24 h of FG treatment (Scale bar: 60 μm; **Bi**) and nuclear co-staining with DAPI (blue), quantified as a % percentage of CD11b ^+^ cells, compared with control **(Bii)**. Data represent the mean ± SEM of 5 defined fields from at least three independent experiments. Significance levels are ^∗∗∗^*p* < 0.001, ^∗∗^*p* < 0.01 compared with relevant control.

Confirmation of a FG-mediated induction of a reactive microglial phenotype, as previously indicated (Adams et al., 2007; Piers et al., 2011) was performed by immunocytochemical analysis of characterized activated microglial markers. Control, unstimulated, primary microglial cultures expressed low levels of the activated microglial marker ED-1 (Figure 2Ai), and > 95% of cells stained positive for IB_4_ (Supplementary Figure S1), suggesting highly purified cultures, in a down-regulated state. In support of FG-mediated activation, ED1 protein expression was significantly enhanced when compared with non-treated control cultures (Figure 2Aii). Furthermore, FG treatment significantly increased the surface expression of its putative receptor, CD11b (Figures 2Bi,ii), induction of which has previously been associated with a phagocytic microglial phenotype (Adams et al., 2007). These findings provide further support for a shift in microglial activation when the cells are exposed to FG, although interestingly whilst LPS evoked inducible nitric oxide synthase (iNOS) expression in the microglia, FG did not, suggesting a further divergence in activation profiles (Figure 2Aiii).

### FG-Mediated Neurotoxicity Is Dependent on Secreted Microglial Factors

**FIGURE 3:**
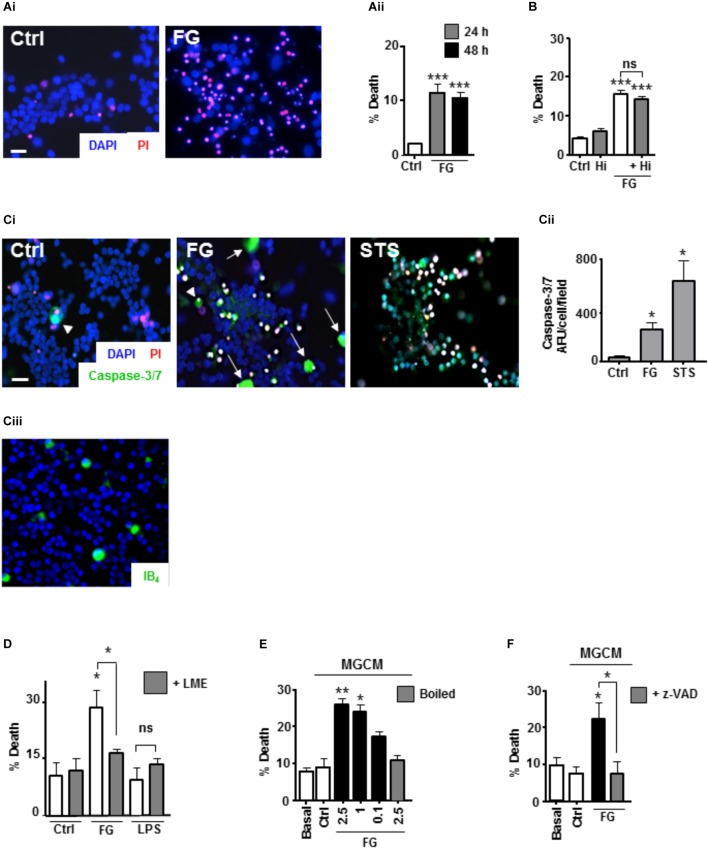
FG-mediated neurotoxicity is dependent on secreted microglial factors. **(A)** Live cell staining with propidium iodide (PI) of ENGCs for analysis of cellular death after FG treatment for 24–48 h (Scale bar: 15 μm; **Ai**), quantified as a percentage of PI ^+^ cells in the population (**Aii**). **(B)** Quantification of cell death in ENGCs after treatment with 2.5 mg/ml FG for 24 h + /– hirudin (40 U/ml). **(C)** Analysis of caspase 3/7 activity by FAM-DEVD-FMK live cell staining in ENGCs following exposure to FG (2.5 mg/ml) or staurosporine (STS, 0.5 μM; Scale bar: 20 μm) for 24 h, **Ci**. Data were quantified and presented as arbitrary fluorescence units (AFU)/cell/field of view; **Cii**). Confirmation of the presence of microglia in ENGCs with IB_4_ cell staining (**Ciii**). All cellular populations were counterstained with Hoechst 33342 (blue). **(D)** Assessment of ENGC death by PI live cell staining after treatment with FG (2.5 mg/ml) or LPS (1 μg/ml) for 24 h before and after microglial ablation ( + LME). **(E)** Analysis of ENGC death by PI live cell staining after exposure to microglial conditioned medium (MGCM) collected from cultures treated for 24 h with FG (0.1–2.5 mg/ml) or untreated (Ctrl). Some MGCM samples from FG treated cultures were boiled to inactivate prior to addition. **(F)** Analysis of ENGC death by PI live cell staining after exposure to MGCM collected from cultures treated for 24 h with FG (2.5 mg/ml) + /– z-VAD-FMK, or untreated (Ctrl). In all graphs, data are the mean ± SEM from at least three independent experiments with internal replicates of at least 3. Significance levels are compared with control condition in each graph unless otherwise indicated, ^∗^*p* < 0.05, ^∗∗^*p* < 0.01, ^∗∗∗^*p* < 0.001.

Whilst it is clear that FG exposure can induce a pro-inflammatory microglial phenotype, it is unclear whether this induced phenotype would have an effect on neuronal viability. To study this, we exposed ENGCs to FG and performed live cell staining with Hoescht/propidium iodide (PI) for total/dead cell analysis, respectively. After 24 h of exposure to FG, the number of PI-positive cells increased significantly when compared with non-treated controls (Figures 3Ai,Aii), which did not increase further after 48 h of exposure (Figure 3Aii). The cleavage of fibrinogen to fibrin by thrombin is a well-defined mechanism in the coagulation cascade (Binnie and Lord, 1993), and thrombin transcripts are present in these cultures when exposed to FG for 48 h (Supplementary Figure S2). Therefore, it was possible that the observed toxicity was due to cleavage of soluble fibrinogen to insoluble fibrin deposits. To clarify this, cultures were co-treated with FG and the thrombin inhibitor hirudin for 24 h. The percentage of PI-positive cells in the FG + hirudin-treated cultures was not significantly different from cultures treated with FG alone (Figure 3B) suggesting soluble FG rather than cleaved fibrin is the cause of the observed reduced neuronal viability. Death was not due to endotoxin contamination of FG since polymyxin B (PMX) treatment to remove endotoxin did not ablate FG-induced death but did ablate LPS induced death (Supplementary Figure S3).

To further characterize the death-signaling pathways induced in the ENGCs by FG exposure, live cell staining for the apoptotic executioner caspase-3/7 was performed, using the fluorescent substrate FAM-DEVD-FMK (Darzynkiewicz et al., 2002). Following 24 h exposure to FG, a significant increase in caspase-3/7 activity was observed, when compared with non-treated control cultures (Figures 3Ci,Cii). Interestingly the most pronounced staining seemed to correlate with cells with large, rounded cytoplasmic morphology, uncharacteristic of neuronal anatomy (white arrow heads, Figure 3Ci), and unlike the prominent staining observed after staurosporine (STS; positive control for the induction of apoptosis) exposure (Figures 3Ci,Cii). These cells fit the description of microglia that we know are present in the ENGCs as confirmed with IB_4_ staining and previously published literature (Figure 3Ciii; Piers et al., 2011; Jebelli et al., 2015). Furthermore, previous studies have suggested that microglial activation is dependent on non-apoptotic caspase-3 activity (Shen et al., 2017). Therefore, we wanted to identify whether microglial caspase-3 activity was important in the observed reduction in neuronal viability. Initially, we performed microglial ablation on ENGCs and found that removal of microglia from the cultures significantly attenuated the observed FG-mediated neuronal death (Figure 3D). Interestingly, LPS mediated neuronal death was not significantly different from control levels, suggesting that whilst the ability of FG and LPS to modulate microglial reactivity is similar in terms of cytokine release, the underlying mechanisms of any subsequent neuronal insults differ (Figure 3D).

Further support for the involvement of microglia in FG-mediated neuronal death, specifically, the secreted factors from microglia, was identified by exposing microglia-depleted ENGCs to conditioned medium from primary microglia cultures (MGCM) exposed to FG (Figure 3E). We observed a significant increase in neuronal death when ENGCs were exposed to MGCM from FG-treated microglial cultures when compared to the addition of control MGCM, which could be attenuated by boiling the MGCM prior to its addition to the ENGCs (Figure 3E). Carry-over of FG in MGCM to exert a direct effect is also unlikely since negligible FG was detected in FG-MGCM (Supplementary Figure S4). Finally, we found it was possible to attenuate the effect of MGCM from FG-treated microglia cultures on ENGCs by pre-incubating the microglia cultures with the caspase-3/7 inhibitor z-VAD-FMK (Figure 3F). MG exposed to FG, or LPS, showed caspase-3 activation but no pyknotic nuclei suggesting non-apoptotic activation of caspase-3 (Supplementary Figure S6). Taken together, these data strongly support a microglial-mediated mechanism for the observed increase in FG-induced neurotoxicity, centered on the release of soluble factors, and suggests a possible role for non-apoptotic activation of caspase-3 in microglia within this mechanism, as our previous investigations found no significant increase in PI-positive (i.e., dead cells) nuclei in primary microglial cultures exposed to FG (Piers et al., 2011).

### FG Induces ER Stress-Associated Neuronal Death via Microglial TNFα Release

**FIGURE 4:**
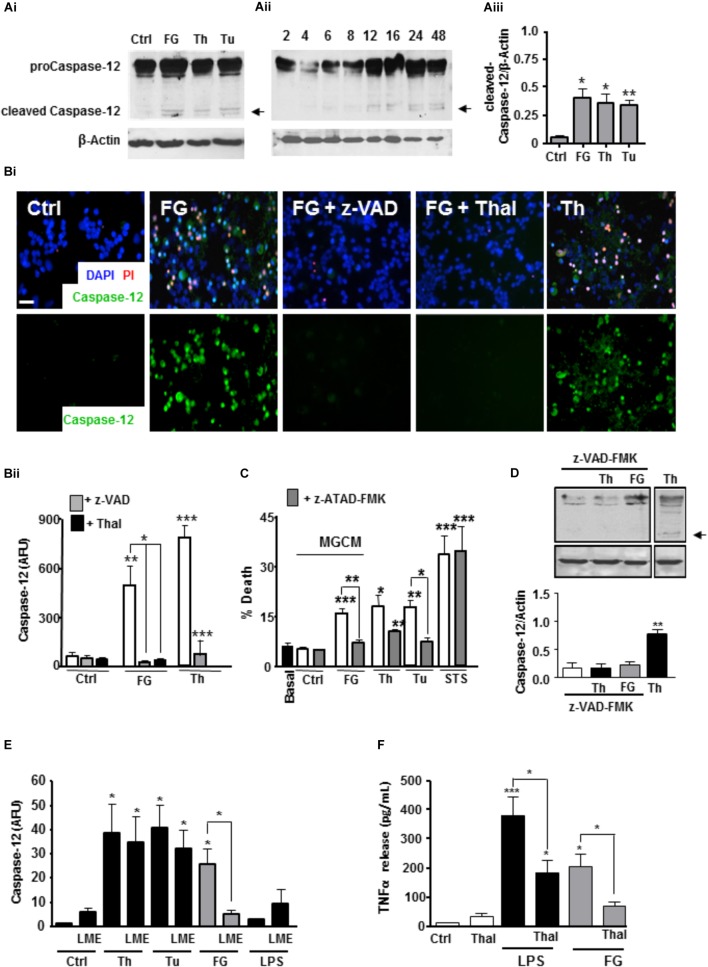
FG inducesneuronal ER stress signaling via microglial TNFα release. **(A)** Expression of pro-caspase 12 (55 kDa) and cleaved active caspase 12 (36 kDa) in ENGCs from control cultures (Ctrl), or cultures treated with FG (2.5 mg/ml), thapsigargin (Th, 2 μM), or tunicamycin (Tu, 1 μg/ml) for 24 h as shown in the representative blot **(Ai)**, and quantified by densitometry of cleaved caspase-12 relative to β-Actin levels **(Aiii)**. **Aii** represents a blot showing time course of expression of cleaved caspase-12 in ENGCs treated with FG (2.5 mg/ml) over time (h). **(B)** Analysis of caspase-12 activity by FITC-ATAD-FMK live cell staining in ENGCs following exposure to FG + /- thalidomide (Thal; 40 μM) or z-VAD-FMK (z-VAD; 1 μg/ml), or thapsigargin (Th, 2 μM) for 24 h. **(Bi)**, Scale bar: 20 μm. Data were quantified and presented as arbitrary fluorescence units (AFU)/cell/field of view; **Bii**). **(C)** Analysis of death in ENGCs by PI live cell staining after exposure to microglial conditioned medium (MGCM) collected from cultures treated FG (2.5 mg/ml) or untreated (Ctrl) + /- direct administration of the caspase-12 specific inhibitor z-ATAD-FMK. Th, thapsigargin, 2 μM, Tu, Tunicamycin, 1 μg/ml Staurosporine (STS; 0.5 μM) was used as a positive control for neuronal death. **(D)** Western blot of cleaved caspase-12 in ENGCs following expose to Thapsigargin (Th, 2 μM) or FG (2.5 mg/ml) in the presence of z-VAD-FMK (1μg/ml) showing inhibition of caspase-12 cleavage (arrow). **(E)** Quantification of the relative active caspase-12 fluorescence intensity/cell in ENGC cultures after treatment with Th (2 μM), Tu (1 μg/ml), FG (2.5 mg/ml), or LPS (1 μg/ml) for 24 h, + /- LME pre-treatment for the depletion of microglia (LME). Data are presented in arbitrary fluorescent units (AFU)/cell/field of view). **(F)** ELISA of TNFα secretion from primary microglial cultures following treatment for 24 h with FG (2.5 mg/ml, or LPS (1 μg/ml), co-treated with thalidomide (Thal; 10 μg/ml). In all graphs, data are the mean ± SEM from at least three independent experiments encompassing 5 separate fields of view or from samples from at least three independent experiments. Significance levels are compared with control condition in each graph unless otherwise indicated, ^∗^*p* < 0.05, ^∗∗^*p* < 0.01, ^∗∗∗^*p* < 0.001.

Based on our microarray and cytokine release data, one candidate for a soluble released factor from microglia that may enhance neurotoxicity is TNFα. Studies suggest inflammatory cytokines including TNFα can induce ER stress signaling (Xue et al., 2005; Denis et al., 2010; O’Neill et al., 2013; Dandekar et al., 2015). Cleavage of the ER-located caspase-12 during ER stress triggers downstream apoptotic pathways and is implicated in neurodegenerative disease models associated with BBB dysfunction (Nakagawa et al., 2000). Therefore, we investigated a possible role for TNFα in an ER stress associated mechanism of neurotoxicity. Initially, we found that exposure of ENGCs to FG induced an increase in activated caspase-12 expression as measured by western blotting for the cleaved form of the protein (Figures 4Ai,Aii,Aiii), to levels comparable with the known ER stress inducers, thapsigargin (a specific inhibitor of the sarco-ER calcium-ATPase, SERCA), or tunicamycin (a blocker of protein glycosylation; Nair et al., 2007; Tu). In support of these findings, live cell staining experiments of ENGCs after FG treatment identified significant increases in caspase-12 activity, comparable with levels observed after thapsigargin treatment (Figures 4Bi,Bii). Furthermore, inhibition of caspase-3/7 with z-VAD-FMK, or specific inhibition of TNFα synthesis by thalidomide treatment, was sufficient to attenuate the increased levels of caspase-12 expression (Figures 4Bi,Bii), supporting the hypothesis that a released factor involved in the induction of neurotoxic ER stress signaling was TNFα. In confirmation of ER stress activation, we found CHOP transcripts were also significantly enhanced in microglia exposed to FG (2.5 mg/ml) or LPS (1 μg/ml) for 24 h or Thapsigargin (2μM, as a positive activator of ER stress) (Supplementary Figure S5).

In support of the critical induction of these signaling cascades in the observed FG-mediated neurotoxicity, MGCM from cultures exposed to FG were unable to induce significant neuronal toxicity in ENGCs pre-treated with the caspase-12 specific inhibitor z-ATAD-FMK (Figure 4C). Inhibition of caspase-3/7 also reduced caspase-12 cleavage in ENGCs, suggestive of an upstream mechanism involving microglial activation (Figure 4D). Concomitantly, removal of microglia from ENGCs prevent FG-mediated induction of caspase-12, further supporting a non-cell autonomous mechanism (Figure 4E). Moreover, thalidomide significantly reduced TNFα secretion from microglia treated with FG (Figure 4F). Together, these findings suggest a signaling cascade initiated by FG-mediated microglial activation leading to a non-cell autonomous neurotoxicity via released TNFα and subsequent induction of neuronal ER stress signaling.

## Discussion

After confirming an immune/inflammatory consequence in microglia after exposure to FG, both through microarray analysis of a microglial cell line and proteome analysis of secreted cytokines in primary cultures, we identified a non-cell autonomous signaling cascade capable of inducing neurotoxicity through a mechanism that depended on microglial-released TNFα and neuronal ER stress. Importantly we were able to pharmacologically inhibit the FG-induced neurotoxicity either by targeting TNFα transcription with the putative inhibitor, thalidomide, or by blocking neuronal caspase-12 activity with the specific inhibitory peptide z-ATAD-FMK. It cannot be discounted that thalidomide may also block transcription of other inflammatory mediators such as IL-6 or that other factors play a role in death cascades induced by FG. For example we show also that IL-6 is secreted by microglia in response to FG. However, thalidomide has been shown to selectively inhibit TNFα secretion from immune cells (Sampaio et al., 1991) strongly suggesting TNFα plays a pivotal role in the inflammatory-linked neurotoxicity observed here. Linkage between increased pro-inflammatory cytokine release, particularly TNFα and IL-6, and the induction of ER stress signaling is supported by others (Xue et al., 2005; Denis et al., 2010; Lourenco et al., 2013; O’Neill et al., 2013). Interestingly elevated systemic fibrinogen, TNFα and IL-6 in mid-life have also been linked with brain shrinkage, cognitive decline, and AD in later life (Marsland et al., 2008; Holmes et al., 2009). Furthermore, ER stress can mediate upregulation of pro-inflammatory cytokines (Chen et al., 2013; Morris et al., 2018) and neuronal ER stress has been linked to inflammation and AD pathology (Salminen et al., 2009). Whilst in rodents, activation of caspase 12 leads to ER stress, in humans, the gene may produce a truncated non-functional protein; caspase 4 in humans is regarded as the homolog for caspase 12 in rodents (Gao and Wells, 2013). Human cell lines have been shown to respond to AD-linked amyloid beta peptides, with ER stress induced caspase-4 activation similarly to the activation of ER stress and subsequent caspase-12 by AD-linked amyloid beta peptides in rodents (Nakagawa et al., 2000). Thus future work on human microglia should take into consideration the role of both caspase 4 (Hitomi et al., 2004) and caspase 12 in fibrinogen-induced activation of microglia.

Taken together our present findings are the first to directly link FG induced release of TNFα to an induction of neuronal ER stress. The current neurotoxicity results are unlikely to be due to a direct FG crossover in the MGCM because blotting of the conditioned medium for FG revealed extremely low levels of the protein, below the effective concentration.

It is of course highly likely that other unidentified factors contribute to the mechanism. Previously we identified that TNFα-induced neuronal death cascades required a cofactor (Taylor et al., 2005), and indeed, Denis et al. (2010) found TNFα alone was unable to induce a full ER stress response. Interestingly, however, TNF inhibitors have been recently proposed as a repurposed therapy for dementias such as AD (Mason et al., 2018). Furthermore, as mentioned above, IL-6 was also secreted in response to FG and elevation of this cytokine has been implicated in a number of neurodegenerative diseases including AD (Rothaug et al., 2016). Increasing evidence links changes in brain parenchymal and plasma levels of fibrinogen with microglial and inflammatory responses in a number of neurodegenerative diseases including multiple sclerosis (Adams et al., 2007; Ryu and McLarnon, 2009; Acuňa et al., 2017) and Alzheimer’s disease (Cortes-Canteli et al., 2012) and following stroke (Luan et al., 2017). Here we present new evidence of a pathway activated in microglia by fibrinogen which has ramifications for neuronal survival and which has not yet been observed in these conditions.

## Conclusion

Our findings suggest a FG-induced TNFα-non-cell autonomous microglial-neuronal ER stress mediated pathway with implications for early signaling cascades activated in inflammatory-mediated dementias such as AD.

## Author Contributions

JP and EE conceived the project. JP, EE, and TP designed the experiments. TP, EE, CV-L, and IS carried out the experiments. TP, EE, CV-L, andMMcarried out the data analysis. JH facilitated genome data analysis. JP and JH obtained the funding. TP, EE, and JP wrote the paper.

## Conflict of Interest Statement

The authors declare that the research was conducted in the absence of any commercial or financial relationships that could be construed as a potential conflict of interest.
