# Supplementary figures and images for: Soluble Fibrinogen Triggers Non-cell Autonomous ER Stress-Mediated Microglial-Induced Neurotoxicity
Source: Front Cell Neurosci. 2018 Nov 19;12:404. doi: 10.3389/fncel.2018.00404 (PMC6257202; doi:10.3389/fncel.2018.00404)

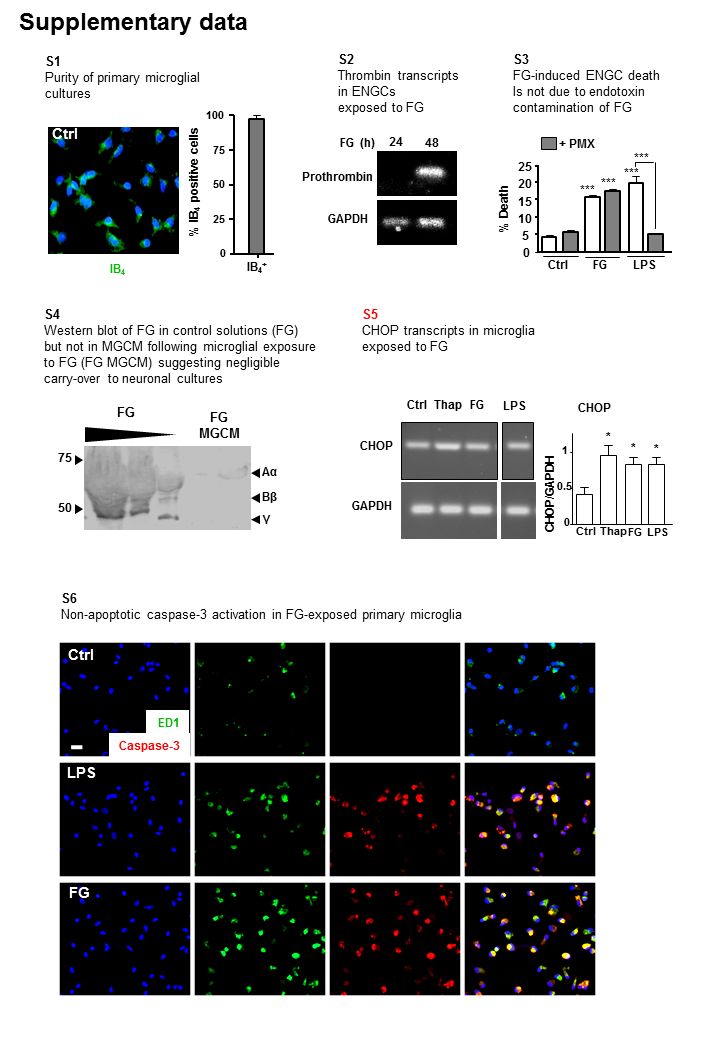

Supplement: Supplementary file 1 [file Image_1.TIF]
